# Pain in experimental autoimmune encephalitis: a comparative study between different mouse models

**DOI:** 10.1186/1742-2094-9-233

**Published:** 2012-10-06

**Authors:** Jianning Lu, Martina Kurejova, Laura N Wirotanseng, Ralf A Linker, Rohini Kuner, Anke Tappe-Theodor

**Affiliations:** 1 Pharmacology Institut, University of Heidelberg, Im Neuenheimer Feld 366, Heidelberg, D-69120, Germany; 2 Department of Neurology, Universitätsklinikum Erlangen, Schwabachanlage 6, Erlangen, D-91054, Germany

## Abstract

**Background:**

Pain can be one of the most severe symptoms associated with multiple sclerosis (MS) and develops with varying levels and time courses. MS-related pain is difficult to treat, since very little is known about the mechanisms underlying its development. Animal models of experimental autoimmune encephalomyelitis (EAE) mimic many aspects of MS and are well-suited to study underlying pathophysiological mechanisms. Yet, to date very little is known about the sensory abnormalities in different EAE models. We therefore aimed to thoroughly characterize pain behavior of the hindpaw in SJL and C57BL/6 mice immunized with PLP_139-151_ peptide or MOG_35-55_ peptide respectively. Moreover, we studied the activity of pain-related molecules and plasticity-related genes in the spinal cord and investigated functional changes in the peripheral nerves using electrophysiology.

**Methods:**

We analyzed thermal and mechanical sensitivity of the hindpaw in both EAE models during the whole disease course. Qualitative and quantitative immunohistochemical analysis of pain-related molecules and plasticity-related genes was performed on spinal cord sections at different timepoints during the disease course. Moreover, we investigated functional changes in the peripheral nerves using electrophysiology.

**Results:**

Mice in both EAE models developed thermal hyperalgesia during the chronic phase of the disease. However, whereas SJL mice developed marked mechanical allodynia over the chronic phase of the disease, C57BL/6 mice developed only minor mechanical allodynia over the onset and peak phase of the disease. Interestingly, the magnitude of glial changes in the spinal cord was stronger in SJL mice than in C57BL/6 mice and their time course matched the temporal profile of mechanical hypersensitivity.

**Conclusions:**

Diverse EAE models bearing genetic, clinical and histopathological heterogeneity, show different profiles of sensory and pathological changes and thereby enable studying the mechanistic basis and the diversity of changes in pain perception that are associated with distinct types of MS.

## Background

Multiple sclerosis (MS) is one of the most common neurological diseases mostly affecting young adults. It is an incurable, chronic inflammatory, progressive neuroinflammatory and neurodegenerative disease with a still unclear etiology. Among others, pain is one of the critical MS symptoms. While research on pain in MS is performed with increasing frequency, the literature remains ambiguous to date. Many studies are based on questionnaires and the reports on pain prevalence in MS patients vary from 29% [1] up to 86% [2]. Some studies report no difference in the frequency of pain in MS patients compared to the background population, but report a higher intensity and impact of pain on daily life in MS patients [3]. It has been reported that 32% of patients indicate pain among the most severe symptoms of MS [4], and 12% of various pain syndromes are even classified as the worst symptom of the MS itself [5]. Symptoms of neuropathic pain, including mechanical or cold allodynia as well as thermal and mechanical hyperalgesia have been described [6-9]. Chronic pain in MS severely reduces the quality of the patient’s life and therefore deserves detailed analysis. So far, not much is known about the mechanisms underlying MS-related pain and its treatment remains difficult. Therefore, there is a major and unmet need for basic research on molecular mechanisms underlying the development and chronicity of pain in MS.

Various animal models mimicking the disease have been used for decades, the most prevalent being experimental autoimmune encephalomyelitis (EAE), which closely resembles MS [10]. The use of diverse immunogenic peptides against central nervous system (CNS) components in the EAE model enables simulation of diverse types of MS (for example, relapsing-remitting, progressive, *etcetera*). A major difference between MS and EAE is that whereas MS is a spontaneous disease, EAE has to be artificially induced using strong immune adjuvants. Only particular combinations of antigen and rodent strain can elucidate EAE [11,12], leading to specific disease profiles [11-14]. Moreover, EAE is studied mainly in inbred strains; hence, the genetic heterogeneity which is critical in the MS populations is only reflected when different models of EAE are studied in parallel [11].

Pain hypersensitivity of the hindpaw has been previously reported in mouse EAE models [15-18]. However, a comprehensive temporal analysis and comparison thereof in different models representing different subtypes of MS has been missing so far. In this study, we sought to comprehensively analyze nociceptive sensitivity during the whole disease course in two different EAE mouse models, namely SJL mice immunized with PLP_139-151_ peptide and C57BL/6 mice immunized with MOG_35-55_ peptide. Moreover, we performed detailed immunohistochemical analyses to address pathophysiological changes that are potentially linked to differences in pain behavior between the two models, and we performed electrophysiological measurements on peripheral nerve terminals. Our results showed that distinct EAE models are associated with specific profiles and temporal courses of changes in pain sensitivity as well as particular patterns of neurochemical changes in the spinal cord.

## Methods

### Animals and induction of experimental autoimmune encephalomyelitis

Female SJL/J mice were purchased from Harlan Laboratories (Borchen, Germany) and C57BL/6 J mice were purchased from Janvier (Le Genest Saint Isle, France). For the induction of EAE, female mice at age eight weeks, received subcutaneous injections in both flanks of either 50 μg MOG_35-55_ peptide or 100 μg PLP_139-151_ peptide (synthesized at German Cancer Research Center; DKFZ, Genomics and Proteomics Core Facilities, Peptide Synthesis, Heidelberg, Germany) in PBS emulsified in an equal volume of complete Freund's adjuvant (CFA) containing *Mycobacterium tuberculosis* H37RA (Difco, Detroit, MI, USA) at a final concentration of 0.5 mg/ml under Isofluran anesthesia. Control mice were immunized with ovalbumin (50 μg) in PBS/CFA. Two injections of pertussis toxin (List Biological Laboratories Inc., Campbell, CA, USA; 200 ng per mouse intraperitoneal) were given on the day of immunization and 48 hours later. Animals were weighed and scored for clinical signs of disease on a daily basis. Disease severity was assessed using a scale ranging from 0 to 10; scores were as follows [19]: 0 = normal; 1 = reduced tone of tail; 2 = limp tail, impaired righting; 3 = absent righting; 4 = gait ataxia; 5 = mild paraparesis of hindlimbs; 6 = moderate paraparesis; 7 = severe paraparesis or paraplegia; 8 = tetraparesis; 9 = moribund; 10 = death. If necessary, food was provided on the cage floor.

### Behavioral nociceptive testing

All animal procedures including the EAE protocol under section:‘Animals and induction of experimental autoimmune encephalomyelitis’ were conducted with the approval of the ethics commitee by the local governing body (Regierungspräsidium Karlsruhe, Germany). All behavioral measurements were done in awake, unrestrained, age-matched female mice. All tests were performed in an appropriate quiet room between 10 am and 4 pm.

Analysis of paw withdrawal latency in response to an infrared beam (which generates a heat ramp) was done as described in earlier publications [20,21] (for example, Plantar test apparatus, Hargreaves' Method, Ugo Basile Inc.). Mechanical sensitivity was tested in the same cohort of animals via manual application of calibrated von Frey hair filaments (0.04 g to 1.4 g) to the plantar surface of the hindpaw as described for earlier studies [20]. The hindpaw withdrawal latency upon heat stimulation using the plantar test apparatus and the hindpaw response to von Frey hair stimulation was assessed every second to third day, alternately.

### Locomotion and exploratory activity

General activity and novelty-induced explorative behavior was measured by using an open field chamber (44 x 44 cm; Ugo Basile, Comerio, Italy) under normal lighting conditions. A video tracking software (ANY-Maze, Ugo Basile, Italy) was used to monitor the mice over ten minutes. The following parameters were analyzed: distance travelled (horizontal activity), speed and immobility time.

### Afferent recordings in skin-nerve preparation

An *in vitro* skin nerve preparation was used to study the properties of mechanosensitive C fibers, two types of Aβ-afferent (slowly adapting fibers (SA) and rapidly adapting fibers (RA)), and Aδ-afferent fibers that innervate the skin of the hind paw. Experiments were performed on the dissected skin of control mice and SJL-EAE mice in the chronic phase of the disease. Animals were killed by CO_2_ inhalation, and the saphenous nerve was dissected with the skin of the dorsal hindpaw attached and mounted in an organ bath *inside-up* to expose the dermis. The preparation was perfused with an oxygen-saturated modified synthetic interstitial fluid solution containing (in mM) 123 NaCl, 3.5 KCl, 0.7 MgSO_4_, 1.5 NaH_2_PO_3_, 1.7 NaH_2_PO_4_, 2.0 CaCl_2_, 9.5 sodium gluconate, 5.5 glucose, 7.5 sucrose, and 10 HEPES at a temperature of 32 ± 1°C and pH 7.4 ± 0.05. Fine filaments were teased from the desheathed nerve, placed in separate chamber, and placed on a recording electrode.

Nerve fibers were classified according to their conduction velocities, von Frey thresholds, and firing properties. Electrical stimulation of the nerve fiber was employed to calculate conduction velocities of individual nerve fibers. Fibers which conducted <1 m/s, fibers conducting between 1 to 10 m/s, and the fibers conducting with the velocity >10 m/s were considered to be unmyelinated C-fibers, myelinated Aδ-fibers and thickly myelinated low threshold mechanoceptors (RA and SA), respectively. The threshold for each unit was tested using calibrated von Frey filaments; the thinnest filament that elicited three action potentials in the time of approximately 2 seconds of pressing the filament on the units was taken as a threshold.

Once the receptive field was identified using the glass rod, a computer-controlled linear stepping motor (Nanomotor Kleindiek Nanotechnik, Reutlingen, Germany) was used to apply standardized mechanical stimuli. Each fiber was tested with a series of displacement mechanical stimuli ranging from 6 to 384 μm for both control and EAE animals. Electrophysiological data were collected with a Powerlab 4.0 system (ADInstruments**,** Spechbach, Germany) and analyzed off-line with the spike histogram extension of the software.

### Immunohistochemistry

Mice were perfused with 0.1 M phosphate buffer saline and 4% paraformaldehyde (PFA). Spinal cords were isolated and post-fixed for up to 16 hours in 4% PFA. Free-floating vibratome sections (50 μm) were processed for immunofluorescence protocol. Sections were incubated for 30 minutes at 80°C in prewarmed 10 mM sodium citrate buffer (pH 8) for antigen retrieval [22] and processed according to standard immunofluorescence protocol. The following antibodies were used: rabbit polyclonal anti-CGRP (Product ID : 24112; 1:200; ImmunoStar Inc., Hudson, WI, USA), Streptavidin-conjugated Isolectin B4 (1:100; Vector laboratories, Burlingame, CA, USA), rabbit polyclonal Iba-1 (Product ID : 019–19741; 1:500; Wako, Richmond, VA, USA), mouse polyclonal anti-GFAP (Product ID : 73–240; 1:200; NeuroMab, Antibodies Incorporated, Davis, CA, USA), mouse monoclonal NeuN (Product ID : MAB377; 1:200; Millipore, Billerica, MA, USA), rabbit polyclonal anti-Fox3 (Product ID : MCA-1B7; 1:500; EnCor Biotechnology, Gainsville, FL, USA).

### Illustrations and densitometry

Fluorescence images were obtained using a laser scanning confocal microscope (Leica TCS AOBS, Bensheim, Germany). For quantitative measurement of microglia and astrocytes, images were obtained in a confocal series over a thickness of 50 μm using the same laser intensity in all images. The fluorescence signal intensity in per unit area was measured densitometrically using NIH ImageJ software (National Institutes of Health, Bethesda, Maryland, USA) Data were averaged from four areas per section and two sections per mouse in groups of at least four animals in three independent experiments.

### Statistics

If not indicated differently, all data are presented as mean ± standard error of the mean (S.E.M.). For comparisons of multiple groups, analysis of variance (ANOVA) for random measures was performed followed by post-hoc Bonferroni’s test, and for the comparison of two groups Student’s *t*-Test was used to determine statistically significant differences. A value of *P* <0.05 was considered to be statistically significant.

## Results

### Disease progression, pain and locomotion

**Figure 1:**
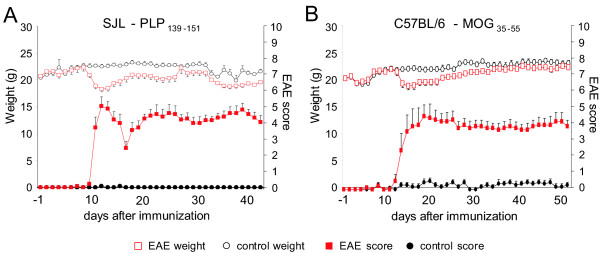
**Mean clinical disease courses and bodyweight.** Female SJL mice (n = 9) were immunized with PLP_139-151_ peptide (**A**) and female C57BL6 mice (n = 7) were immunized with MOG_35-55_ peptide in CFA. (**B**) Control mice (n = 6 respectively) were immunized with ovalbumin in CFA. CFA, complete Freund's adjuvant.

We actively immunized female mice from the SJL and C57BL/6 strains with either the PLP_139-151_ peptide or the MOG_35-55_ peptide (referred to henceforth as SJL-EAE or C57-EAE mice, respectively). Control mice underwent the same immunization protocol using ovalbumin. SJL-EAE mice showed a typical relapsing-remitting disease pattern, whereas C57-EAE mice developed chronic EAE. After immunization, SJL-EAE mice displayed the first signs of disease onset with tail weakness on day 10 and reached a peak in motor deficit functions at day 12 (Figure 1A), whereas C57-EAE mice showed the first symptoms at day 11 and a maximal disease score at day 17 (Figure 1B). As usually seen, EAE mice lost 1 to 2 g of body weight immediately preceding the onset of the disease (Figure 1). The degree of the EAE in the chronic phase was comparable over both models, as indicated by a similar disease score (Figure 1).

**Figure 2:**
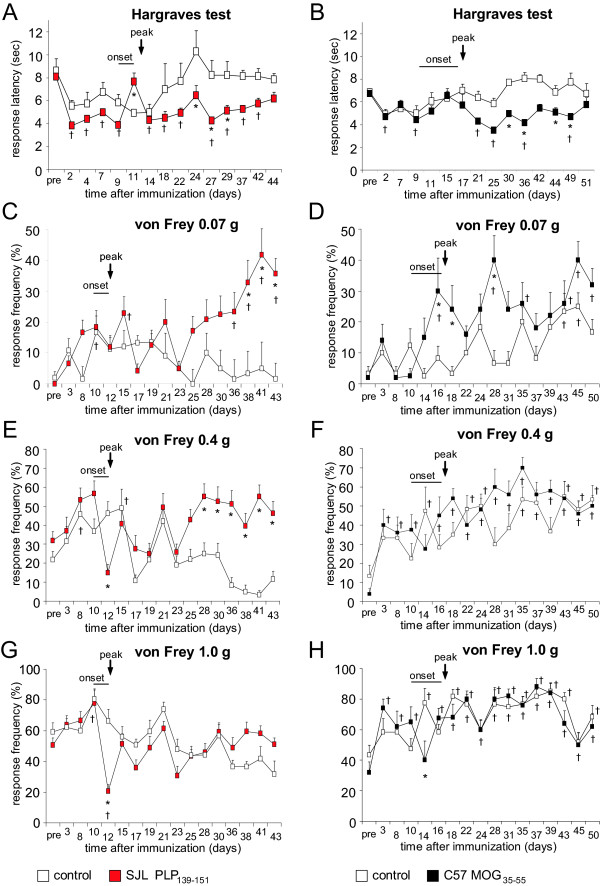
**Analysis of nociceptive sensitivity in SJL mice immunized with PLP**_**139-151**_**peptide, C57 mice immunized with MOG**_**35-55**_**peptide and corresponding control mice.** (**A**, **B**) time-course of withdrawal latency to radiant heat in (**A**) SJL-EAE mice (left column, red symbols) and (**B**) C57-EAE mice (right column, black symbols). (**C**-**H**) Comparison of response frequency to von Frey hair filament stimulation. Response frequency toward the application of 0.07 g von Frey hair filament in (**C**) SJL-EAE and (**D**) C57-EAE mice, 0.4 g von Frey hair filaments in (**E**) SJL-EAE and (**F**) C57-EAE mice, and 1.0 g von Frey hair filament in (**G**) SJL-EAE and (**H**) C57-EAE mice. SJL-EAE mice developed major mechanical allodynia in the chronic phase whereas C57-EAE mice showed minor allodynia in the onset and peak phase. n = 6 mice/ group, *P <0.05 as compared to all control groups, ^†^as compared to basal values within a group, ANOVA, post hoc Bonferroni’s test. All data points represent mean ± SEM. EAE, experimental autoimmune encephalomyelitis.

In addition to monitoring clinical disease symptoms on a daily basis over 44 days (SJL-EAE mice) or 52 days (C57-EAE mice), we investigated nociceptive thresholds in response to heat and mechanical stimuli. We found that the response latency towards heat stimuli dropped significantly in SJL-EAE and C57-EAE mice following immunization as compared to basal response latencies (Figure 2A,B). Mice in both EAE models developed significant thermal hyperalgesia in the chronic phase of the disease (Figure 2A,B; Table 1). Thus, the time course of thermal hyperalgesia was not different across the two models.

**Table 1:** Summary and overview of the main characteristics of SJL PLP139-151 peptide immunized mice and C57 MOG35-55 peptide immunized mice

**Parameter**	**SJL-PLP**_**139-151**_	**C57-MOG**_**35-55**_
**Thermal hyperalgesia**		
Onset	+	∅
Peak	+	∅
Chronic	++	+
**Mechanical allodynia**		
Onset	∅	(+)
Peak	∅	(+)
Chronic	++	∅
**Microglia activation**		
Onset	+	+
Peak	+++	++
Chronic	++	+
**Astrocyte activation**		
Onset	+	++
Peak	++	++
Chronic	+++	++

Behavioral and immunohistochemical characteristics indicate that SJL-EAE mice develop much more pain and show stronger microglia and astrocyte activation.

**Figure 3:**
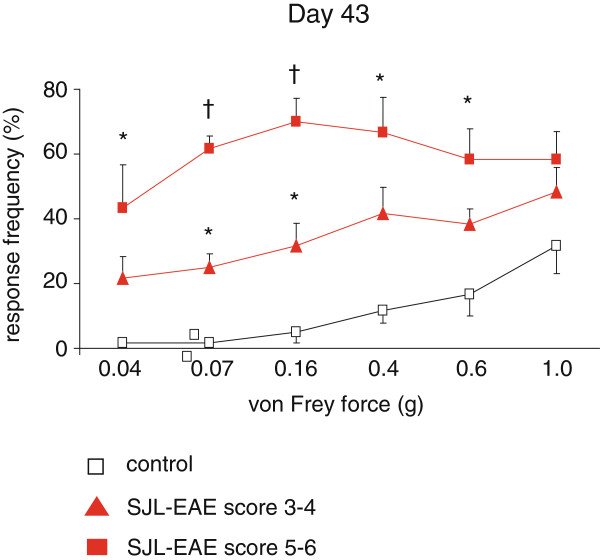
**Comparison of response frequency to von Frey hair filament stimulation in the chronic phase of the EAE (day 43).** SJL-EAE mice with a higher disease score (score 5 to 6) show more pronounced mechanical allodynia than SJL-EAE mice with a lower score (score 3 to 4) as compared to control mice. n = 6 mice/ group, *P <0.05 as compared to control mice at this measuring point, ^†^as compared to all other groups at this particular measuring point, ANOVA, post hoc Bonferroni’s test. All data points represent mean ± SEM. EAE, experimental autoimmune encephalomyelitis.

We applied mechanical pressure via von Frey hair filaments (0.04 g to 1.4 g force) to the plantar surface of the hindpaws. The application of low magnitude of forces (von Frey filaments of forces between 0.04 g to 0.07 g), which do not normally evoke nociceptive withdrawal in control mice, elicited withdrawal in SJL-EAE mice in the chronic phase of the disease starting from day 36 onwards and lasting over the whole period of investigation (data with 0.07 g force are shown in Figure 2C). The same stimulus also elicited withdrawal behavior in C57-EAE mice but in a different temporal time frame: in the onset and peak phase of the disease (Figure 2D). The application of more intense forces to the plantar surface of the paw (von Frey hair filaments between 0.16 g to 0.6 g), that normally evoke mild nociceptive withdrawal in control mice, resulted in a significant increase in withdrawal response frequency in SJL-EAE mice in the chronic phase of the disease, starting from day 28 after immunization and continuing over the whole observation period (data with 0.4 g force are shown in Figure 2E), whereas the withdrawal behavior of C57-EAE mice did not differ from control mice (Figure 2F). Moreover, we found that mechanical allodynia correlated with the clinical scores. SJL-EAE mice with higher clinical scores (score 5 to 6) showed a more pronounced mechanical allodynia than EAE mice with moderate symptoms (score 3 to 4) (Figure 3). Interestingly, the paw withdrawal response frequency towards the application of von Frey filaments of stronger force (1 g or 1.4 g) was comparable between either SJL-EAE mice and control mice (data with 1.0 g force are shown in Figure 2G) or C57-EAE mice and controls (Figure 2H). This shows that SJL-EAE mice develop nociceptive mechanical allodynia in the chronic phase of the disease. The differences in the behavioral phenotypes are summarized in Table 1.

**Figure 4:**
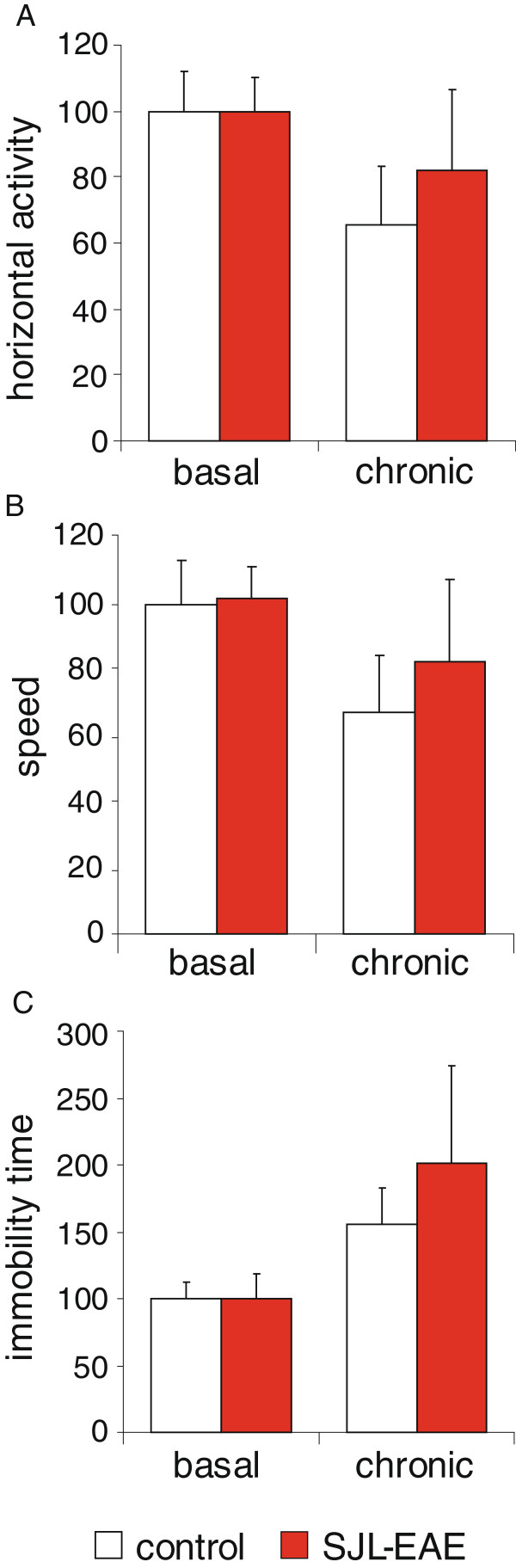
**Behavioral and motor task analysis of SJL-PLP**_**139-151**_**peptide immunized mice and control mice in the open field apparatus.** Parameters were analyzed before immunization (basal) or in the chronic phase of the EAE (day 38) and represented as percent change over basal values. (**A**) the distance of travelling is represented as horizontal activity, (**B**) mean speed of movement, (**C**) immobility time. SJL-EAE mice do not show any deficits in motor performance. n = 5 to 7 mice per group. EAE, experimental autoimmune encephalomyelitis.

Intrigued by the marked mechanical hypersensitivity in the chronic phase of EAE in SJL mice, we questioned whether their locomotor activity would be altered. Using the open field test apparatus SJL-EAE mice did not demonstrate any difference in horizontal activity when compared to either the control mice or to their basal behavior before the induction of EAE (Figure 4A). Additional parameters, as movement speed (Figure 4B) or immobility time (Figure 4C) were not different between EAE and control animals in the chronic phase of the disease or as compared to basal behavior.

Thus, SJL-EAE mice did not reveal aberrant behavioral changes associated with EAE despite the presence of nociceptive hypersensitivity to sensory stimuli.

### Electrophysiological analyses of peripheral nerve activity

**Figure 5:**
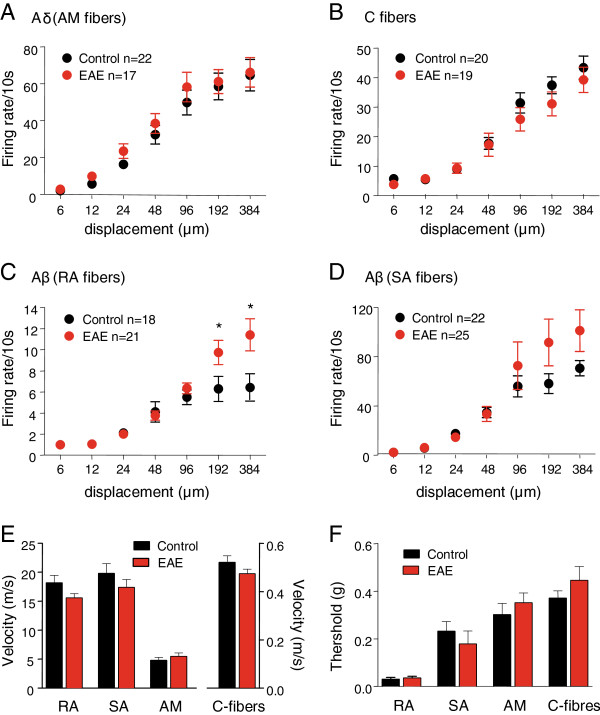
**Electrophysiological analysis of excitability of peripheral afferent fibers in the skin nerve preparation in SJL-PLP**_**139-151**_**peptide immunized mice and control mice.** Shown are electrophysiological recordings of firing rates evoked by application of increasing 10 seconds lasting pressure via a nanomotor (expressed in terms of displacement) from (**A**) Aδ-type of mechanonociceptors, (**B**) C-mechanonociceptors (C-fibers), (**C**) Aβ low threshold mechanoceptors, rapidly adapting (RA), and (**D**) slowly adapting (SA), in the skin-nerve preparation derived from the paws of control mice (black symbols) and SJL-EAE mice (red symbol) in the chronic phase of the disease (days 35 to 45). (**E**) Proximal nerve fiber velocities were calculated from the action potential latency to the electrical stimulation and distance of the receptive field from the measuring electrode. (**F**) Mechanical threshold to the accidently applied calibrated von Frey filaments. *indicates significant statistical difference (*P* <0.05; ANOVA followed by post-hoc Bonferroni’s test). n represents the number of fiber type for each tested animal group. EAE, experimental autoimmune encephalomyelitis.

In order to characterize the firing properties of peripheral afferents in the chronic phase of the disease, the skin nerve preparation of the saphenous nerve was employed on eight SJL-EAE mice and seven control mice in the chronic phase of the disease (day 35 to 45) (Figure 5). Firing properties of four different fiber types innervating the hindpaw were investigated in response to graded mechanical stimuli, namely mechanosensitive C-fiber nociceptors, Aδ mechanonociceptors, SA, and RA low-threshold Aβ mechanoceptors, which were identified on the basis of stimulation as well as conduction and firing properties. Stimulus–response functions of C-fibers and Aδ mechanonociceptors from control and SJL-EAE mice demonstrated no significant changes in the responsiveness to mechanical stimulation (Figure 5A, 5B). Low-threshold SA and RA Aβ fibers isolated from the SJL-EAE animals showed a slight or even statistically significant increase in responses to higher stimulus intensities. Additionally RA and SA low-threshold Aβ fibers and non-myelinated C-fibers (Figure 5E) showed a slight decrease in conduction velocity. There were no changes in mechanical thresholds of different afferent fibers (Figure 5F). So, the functional properties of the nerve fibers in the chronic phase of the EAE are unaltered and unlikely to contribute to the sensory abnormalities.

### Immunohistochemistry on the spinal cord

We investigated lumbar spinal cord section of SJL-EAE mice and control immunized mice at different time points during EAE for the expression of different pain- or EAE-related markers. Because not only white matter abnormalities but also grey matter abnormalities are a basic phenomenon in EAE, we investigated the expression of various key marker proteins at 2 to 3 days after immunization (‘pre’ time point), at disease onset, at peak and in the chronic phase of the disease (day 35 to 45 after EAE induction).

**Figure 6:**
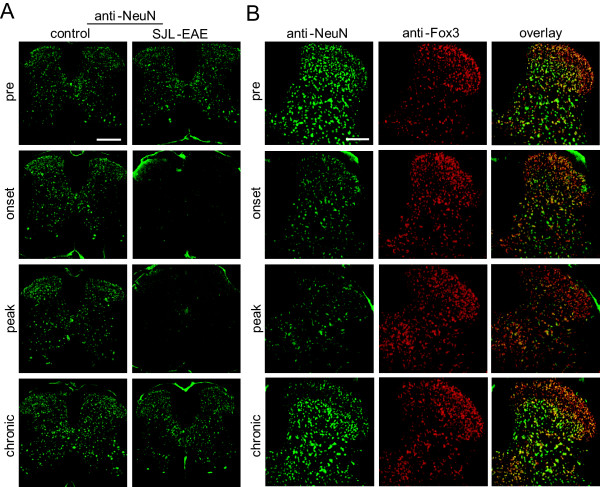
**Expression of NeuN and Fox3 in lumbar spinal cord in SJL-PLP**_**139-151**_**peptide immunized mice and control mice over the time course of the EAE.** (**A**) Typical examples of NeuN expression in spinal cord sections of control immunized mice and SJL-EAE mice at 2 to 3 days following immunization (pre-timepoint), in the onset phase, in the peak phase and in the chronic phase of the disease (day 35 to 40 after immunization) showing a strong decrease in NeuN expression. Scale bar represents 1 mm. (**B**) Examples of dorsal horn spinal cord sections of SJL-EAE mice showing the expression of NeuN (green), Fox3 (red) and the corresponding overlay pictures, indicating a decrease of NeuN antigenicity rather than cellular loss. Scale bar represents 200 μm. EAE, experimental autoimmune encephalomyelitis.

We found a downregulation of NeuN expression throughout the whole spinal cord at disease onset and in the peak phase and an almost complete recovery of NeuN immunogenicity in the chronic phase as compared to control mice (Figure 6A). Recently, NeuN has been identified as the Fox-3 gene product [23]. Therefore, we performed co-labeling of anti-NeuN with anti-Fox-3 antibody. Interestingly, we did not find any difference in Fox-3 expression during the time course of the EAE (Figure 6B), indicating no alteration in the amount of neuronal cells during the time course of the EAE. The loss of NeuN immunoreactivity might be accompanied with specific changes in the EAE disease that lead to a change in NeuN antigenicity, as has been reported in other conditions [24,25].

**Figure 7:**
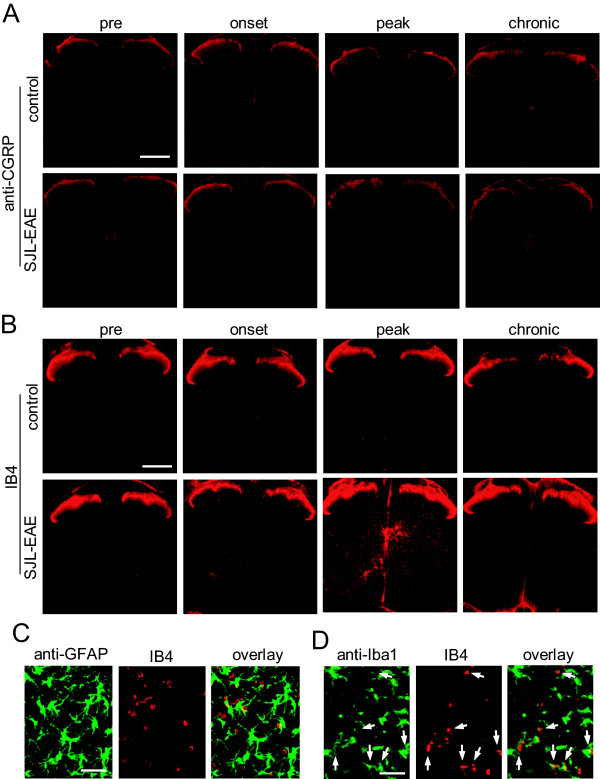
**Spinal patterning of peptidergic (CGRP positive) and non-peptidergic (Isolectin B4 binding) terminals in the lumbar spinal cord of control immunized and SJL-PLP**_**139-151**_**immunized mice over the time course of the EAE.** (**A**) Examples of CGRP expression at 2 to 3 days following immunization (pre-timepoint), in the onset phase, in the peak phase and in the chronic phase of the disease (day 35 to 40 after immunization). (**B**) Examples of Isolectin B4 (IB4) binding at 2 to 3 days following immunization (pre-timepoint), in the onset phase, in the peak phase and in the chronic phase of the disease (day 35 to 40 after immunization). There was an increase in IB4 positive binding throughout the spinal cord in SJL-EAE mice in the onset and peak phase of the disease. (**C**) Co-labeling of anti-GFAP with IB4 revealed no overlay. (**D**) Co-labeling of anti-Iba1 with IB4 showed strong colocalization. Examples are indicated with arrows. Scale bars represent 1 mm in panel A and B and 50 μm in panel C and D. CGRP, calcitonin gene-regulated peptide; EAE, experimental autoimmune encephalomyelitis; IB4, nonpeptidergic isolectin B4.

Additionally we analyzed the patterning of the neuropeptide calcitonin gene-regulated peptide (CGRP) and the nonpeptidergic isolectin B4 (IB4). Although there was no difference in the density of CGRP-immunoreactive fibers in the spinal dorsal horn in SJL-EAE mice or control mice during the time course of the EAE (Figure 7A), we observed an increase in IB4-positive signals throughout the whole spinal cord at the onset of the disease (Figure 7B). We registered maximal increase in IB4 expression at the peak stage of the disease, which decreased in the chronic phase (Figure 7B). Because IB4 selectively binds activated microglia cells [26], our results indicate a strong activation of microglia in SJL-EAE mice at disease onset and at peak phase of the disease. Co-labeling studies with anti-GFAP, a marker for astrocytes and anti-Iba1, a marker for microglia cells, confirmed the expression of IB4 specifically in microglia.

**Figure 8:**
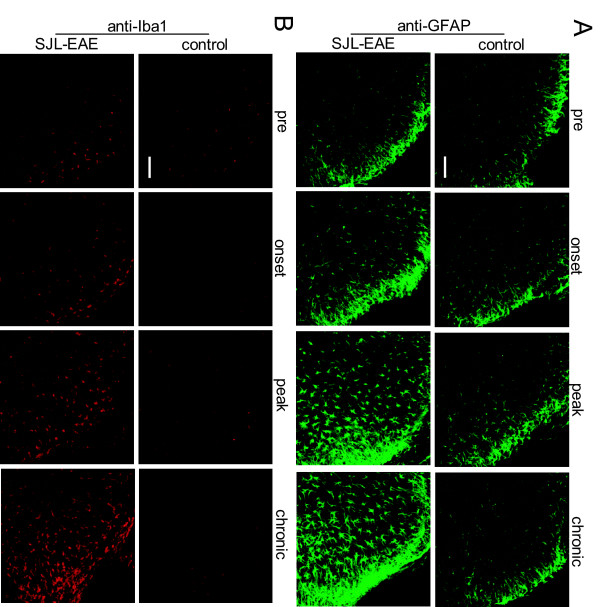
**Astrocyte and microglia expression in the spinal dorsal horn of control immunized mice and SJL-PLP**_**139-151**_**peptide immunized mice over the time course of the EAE. **(**A**) Representative examples of GFAP expression at 2 to 3 days following immunization (pre-timepoint), in the onset phase, in the peak phase and in the chronic phase of the disease (day 35 to 40 after immunization). There was a significant increase in GFAP positive cells over time in SJL-EAE mice. (**B**) Representative examples of Iba1 positive cells at 2 to 3 days following immunization (pre-timepoint), in the onset phase, in the peak phase and in the chronic phase of the disease (day 35 to 40 after immunization). The expression of Iba1 increased significantly over time in SJL-EAE mice in the spinal dorsal horn. Scale bars represent 100 μm in panel A and B. EAE, experimental autoimmune encephalomyelitis.

As glia cells play an important role in EAE we investigated the time course of astrocyte and microglia activity in the spinal cord of SJL-EAE and control mice. Immunohistochemistry with anti-GFAP antibody showed an increase in GFAP-positive cells at disease onset in the spinal dorsal horn (Figure 8A). The number of GFAP positive cells further increased in the peak and chronic phase of the disease, and cells became activated as seen by their morphological changes (Figure 8A). Similarly, using the microglia specific anti-Iba1 antibody, we saw an induction of microglia cells at disease onset and in the chronic phase of the disease and activation of microglia, which was evident by morphological changes (Figure 8B).

**Figure 9:**
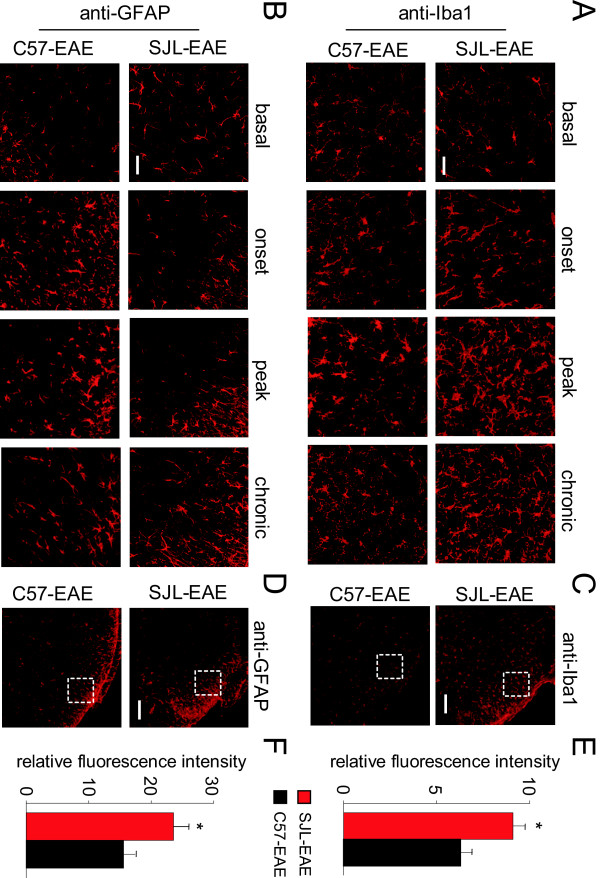
**Analysis and quantification of astrocyte and microglia activation in SJL-PLP**_**139-151**_**peptide immunized mice and C57-MOG**_**35-55**_**peptide immunized mice.** High magnification of lumbar spinal dorsal horn in SJL-EAE and C57-EAE mice at basal level (before immunization), in the onset phase, in the peak phase and in the chronic phase (day 35 to 40 after immunization) of the disease stained with anti-Iba1 (**A**) or anti-GFAP (**B**) antibody. SJL-EAE mice showed a stronger activation of Iba1 and GFAP positive cells than C57-EAE mice. Scale bars represent 25 μm in panel A and B. (**C**,**D**) Overview of the spinal dorsal horn of SJL-EAE and C57-EAE mice in the chronic phase of the disease showing higher immunoreactivity for Iba1 (C) and GFAP (D). Boxed areas indicate where pictures in panel A and B were taken. Scale bars represent 100 μm in panel C and D. (**E**,**F**) Quantification of relative levels of the expression of Iba1 (E) and GFAP (F) proteins in the spinal dorsal horn of SJL-EAE and C57-EAE mice in the chronic phase of the disease. There was a significant higher expression of Iba1 positive cells (E) and GFAP positive cells (F) in SJL-EAE mice as compared to C57-EAE mice. ; EAE, experimental autoimmune encephalomyelitis.

Because microglia and astrocyte activation plays an important role in pain, we compared the time course of microglia and astrocyte activation in SJL-EAE and C57-EAE animals in more detail. Interestingly, we found a comparable activation of microglia as shown with anti-Iba1 antibody in the dorsal horn of the spinal cord during the onset phase in SJL-EAE and C57-EAE mice (Figure 9A), but to a lesser extent in C57-EAE mice as compared to SJL-EAE mice in the peak phase as well as in the chronic phase of the disease (Figure 9A).

To quantify the amount of microglia cells in the chronic phase of the disease, we measured the fluorescence intensity in lamina I and II of the spinal dorsal horn and found a significantly higher fluorescence intensity for Iba1 in SJL-EAE mice as compared to C57-EAE mice (see Figure 9C for example, Figure 9E for quantification). Additionally we compared the expression profile of astrocytes by using an anti-GFAP antibody. We found a stronger activation of astrocytes in C57-EAE as compared to SJL-EAE mice in the onset phase of the disease (Figure 9B). Interestingly, there was an accumulation of GFAP-positive cells in the superficial spinal dorsal horn of SJL-EAE mice in the chronic phase of the disease as compared to C57-EAE mice (Figure 9B). Quantification of the GFAP fluorescence intensity in the spinal dorsal horn revealed a significantly stronger activation of astrocytes in SJL-EAE mice as compared to C57-EAE mice in the chronic phase of the disease (see Figure 9D for example, Figure 9F for quantification).

The differences of microglia and astrocyte activation in the spinal dorsal horn between the two EAE models are summarized in Table 1.

## Discussion

Clinically significant pain is a severe and debilitating symptom associated with MS, however, to date we are far beyond understanding the mechanisms underlying MS-related pain. Animal models mimicking diverse aspects of the disease have been used for decades to study pathological features of the disease and more recently to investigate behavioral changes with respect to pain hypersensitivity.

Chronic pain symptoms in MS are very complex and diverse and could even be indirectly related to MS (reviewed in [27,28]). Pain symptoms, the number of pain sites, and their severity vary among the patients and are often unrelated to the duration of MS [29]. Pain has been reported at the onset of the disease [4] or even as an initial symptom of MS [30]. Pain syndromes are described as increasing with the age of patients and the disease progression [2,4,31], but in most MS studies chronic pain was found to have no significant correlation to age, disease duration or disease course [29,32-37]. Taking this into account, the use of animal models to study MS-related chronic pain syndromes is very limited. We aimed to investigate the sensory properties of the hindpaws as readout for hyperalgesia and allodynia, which constitute one component of MS-related pain

Here, we provide a thorough investigation of nociceptive sensitivity of the hindpaw in two different mouse EAE models over a complete time course of the disease. Additionally, we provide substantiated underlying mechanistical analysis with detailed immunohistochemical data. We found that SJL mice immunized with PLP_139-151_ peptide and C57 mice immunized with MOG_35-55_ peptide clearly showed thermal hyperalgesia, whereas only SJL-EAE mice developed marked mechanical allodynia in the chronic phase of the disease. C57-EAE mice developed mechanical allodynia exclusively towards very low-intensity stimuli during disease onset and peak phase. Our findings are in line with a study from Aicher *et al.*[15] who showed thermal hyperalgesia in SJL-PLP_139-151_ EAE mice in the chronic phase of the disease [15]; however, this was found on the tail and forepaw of the mice. Additionally Olechowski *et al.*[16] and Rodrigues *et al.*[17] reported hindpaw mechanical allodynia and hypernociception before and around the onset phase of EAE in C57-MOG_35-55_ mice [16,17]. Our findings are supported from these studies and clearly demonstrate differences in the sensory properties between the two commonly used EAE models. The use of the same behavioral tests over a long-lasting investigation period under similar conditions enabled us to directly compare the sensory profile of both EAE models.

Pain in MS patients is very diverse and one EAE model cannot mirror the heterogeneity of the disease [11] research perspective should therefore be focused towards the understanding that one EAE -pain model is not sufficient to study MS-related pain. Moreover, depending on the immunization peptides used and their representation in peripheral nervous system [38], peripheral pain may also add to the mechanism of increased pain in neuroinflammation, especially in models of autoimmune neuritis [39,40].

We found a strong activation of glia cells in the spinal dorsal horn in SJL-EAE and C57-EAE mice. This glia activation occured to a different magnitude and over a different time course in both models, that matched the temporal profile of nociceptive hypersensitivity. It is known that microglia and astrocytes are critical players in the effector phase of EAE and MS [41,42] because there is a marked activation of glia cells in both the spinal cord and brain over the course of the disease [43,44]. We hypothesize that the time course and extent of microglia and astrocyte activation in SJL-EAE mice as compared to C57-EAE mice and the subsequent release of diverse signaling molecules constitute the marked differences in the development and maintenance of chronic pain. This theory is supported from a study of Olechowski *et al.*[16], suggesting inflammation and reactive gliosis as key mediators of allodynia in C57-MOG_35-55_ EAE mice [16]. Activated glia cells not only undergo phenotypic changes, which are characterized by altered morphology, but also release a large variety of different signaling molecules, including inflammatory cytokines and chemokines [45-50], which are strongly implicated in pain facilitation [51-55].

There is a large variety of molecules and mediators, and thus, diverse signaling scenarios are possible. Temporally regulated key signaling mediators that possibly account for the development and maintenance of chronic pain in EAE include regulated glial factors such as those that comprise the chemokine monocyte chemoattractant protein-1 (MCP-1), which is released from glia cells and can attract various cell types involved in inflammation and also pain. Previous studies have demonstrated the expression of MCP-1 in the CNS of patients with MS [56-58] or EAE mice [59]. Additionally, the MCP-1 receptor CCR2 has been shown to be critical for the induction of EAE [60]. Accumulating evidence indicates that MCP-1 plays a critical role in chronic pain facilitation via CCR2 receptors [61-64]. Spinal MCP-1 can lead to neuropathic pain behavior [65,66] and induces to the phosphorylation of the mitogen-activated protein kinase (MAPK) extracellular regulated kinase (ERK) [65] in the spinal cord. In addition, Shin *et al.*[67] found a significant increase of different MAPK (phosphorylated ERK, c-jun N-terminal kinase (JNK) and p38) in the rat spinal cord at the peak stage of EAE [67]. The activation of ERK is known to play an important role in central sensitization [68], and JNK has been shown to be persistently activated in spinal cord astrocytes after nerve injury [69,70]. Moreover MCP-1 has been shown to amplify excitatory glutamatergic currents [65] and inhibits GABA-induced currents [71]. Thus, MCP-1 is strongly involved in mechanisms of chronic pain.

Another example is matrix metalloproteinases (MMPs), which are known to be largely implicated in MS and EAE progression [72,73]. A variety of MMPs are upregulated in the spinal cord of EAE mice, among which are MMP-2, MMP-7, MMP-8 and MMP-9 [74-76]. Dong *et al.*[77] recently reported concordant elevated expression of MMP-2 and MMP-9 to a different extent in different EAE models [77]. Moreover, MMP-9 plays an important role in neuropathic pain conditions [78,79] as well as in MS [80-83]. Additionally, the administration of MMP inhibitors or genetical ablation of MMPs reduces the disease severity in different EAE murine models [84-87].

To further support our theory, another mechanistical possibility might be via proinflammatory cytokines (for example, IL-1beta, IL-6 and TNFalpha), which have been shown to lead to the phosphorylation of CREB [79]. CREB is essential for the maintenance of long-term plasticity in dorsal horn neurons [79] and thereby plays an essential role in pain sensitization [79,88-90]. Kim *et al.* suggests that increased phosphorylation of CREB in sensory neurons in the dorsal horns might be involved in the generation of neuropathic pain in EAE [91]. Taken together, there are various signaling pathways arising from activated glia cells which may thereby contribute to pain in EAE and possibly also to MS.

Given that neuro-immune interactions play a critical role in other pain states and given that peripheral immune function is also changed in MS patients [7] it is possible that peripheral neuro-immune interactions contribute to MS-induced pain. In order to clarify potential changes in the peripheral nervous system in SJL-EAE mice, we investigated the electrophysiological properties of peripheral afferent fibers in EAE mice using the skin nerve preparation. EAE is known to cause central demyelination, but there is weak evidence for a peripheral component to the disease [92,93]. In case of a peripheral demyelination one would expect a decrease in velocity of the signal transduction of myelinated Aβ and Aδ fibers. Pender *et al.* observed an impaired response to noxious mechanical stimuli potentially associated with a demyelination-induced conduction block in the small diameter myelinated afferent (Aδ) fibers in the dorsal root ganglia (DRGs) of rabbits or rats with EAE [94-96]. We observed a slight decrease in conduction velocity in myelinated Aβ mechanonociceptors but the observed changes in the peripheral afferents are very mild, indicating only minor peripheral contribution to the disease phenotype which might arise from a different mechanism than possible peripheral demyelination processes.

In summary we show clear differences in pain behavior between different EAE mouse models, which may reflect the heterogeneity in human MS. Moreover the observed differences in glia cell activation most likely contribute to the different pain behavior. This study suggests that microglia and astrocytes represent a good target to investigate pain mechanisms in different EAE mouse models. Future studies would be necessary to elucidate differences in downstream signaling cascades in the different EAE models.

## Conclusions

In summary we show clear differences in pain behavior between different EAE mouse models, which may reflect the heterogeneity in human MS. Moreover the observed differences in glia cell activation most likely contribute to the different pain behavior. This study suggests that microglia and astrocytes represent a good target to investigate pain mechanisms in different EAE mouse models. Future studies would be necessary to elucidate differences in downstream signaling cascades in the different EAE models.

## Abbreviations

CFA: Complete Freund’s adjuvant; CGRP: Calcitonin gene-regulated peptide; CNS: Central nervous system; DRG: Dorsal root ganglia; EAE: Experimental autoimmune encephalomyelitis; ERK: Extracellular regulated kinase; IB4: Isolectin B4; JNK: c-jun N-terminal kinase; MAPK: Mitogen-activated protein kinase; MMPs: Matrix metalloproteinases; MS: Multiple sclerosis; PFA: Paraformaldehyde; RA: Rapidly adapting; SA: Slowly adapting.

## Competing interests

The authors have no conflicts of interest.

## Authors’ contributions

JL carried out behavioral and histological experiments and analyzed results. MK performed skin-nerve electrophysiological experiments, analyzed data and provided figure. RIW carried out open field behavioral experiments and analyzed data. RAL and RK provided general support and participated in the design of the study. RAL helped to improve the manuscript. ATT conceived, designed, and coordinated the study and wrote the manuscript. All authors read and approved the final manuscript.
